# Silica Bonded *S*-Sulfonic Acid: A Recyclable Catalyst for the Synthesis of Quinoxalines at Room Temperature

**DOI:** 10.3390/molecules14051915

**Published:** 2009-05-22

**Authors:** Khodabakhsh Niknam, Dariush Saberi, Maleki Mohagheghnejad

**Affiliations:** Department of Chemistry, Faculty of Sciences, Persian Gulf University, Bushehr 75169 Iran

**Keywords:** silica bonded *S*-sulfonic acid, quinoxalines, 1,2-diamino compounds, 1,2-dicarbonyl compounds

## Abstract

The reaction of 3-mercaptopropylsilica (MPS) and chlorosulfonic acid in chloroform afforded silica bonded *S*-sulfonic acid (**SBSSA**), which was used as a catalyst for the room temperature synthesis of quinoxaline derivatives from 1,2-diamino compounds and 1,2-dicarbonyl compounds. The catalyst could be recycled and reused several times without any loss of efficiency.

## 1. Introduction

**Scheme 1:**
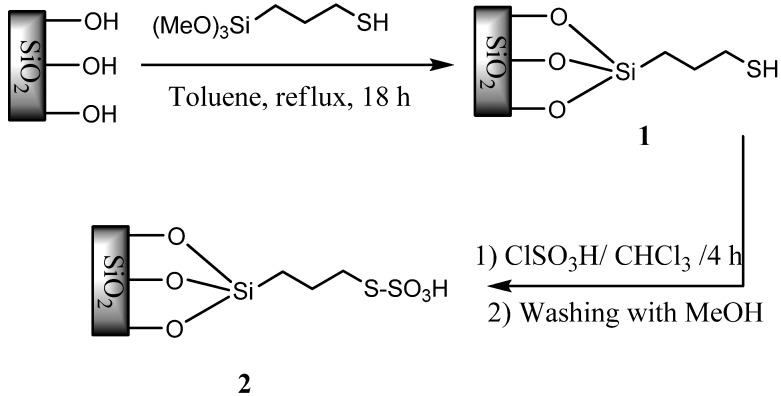
Preparation of silica bonded S-sulfonic acid (**SBSSA**).

In recent years, the search for environmentally benign chemical processes or methodologies has received much attention, and the development of heterogeneous catalysts for fine chemical synthesis has become a major area of research. The potential advantages of these materials over homogeneous systems (simplified recovery and reusability, the potential for incorporation in continuous reactors and micro reactors) could lead to novel environmentally benign chemical procedures for academia and industry [1]. From this viewpoint, a catalytic reaction is a valuable process because the use of stoichiometric reagents that are often toxic poses inherent limitations regarding product purification and waste management from both economical and environmental viewpoints [2]. Application of solid acids in organic transformation has an important role, because they have many advantages such as ease of handling, decreased reactor and plant corrosion problems, and more environmentally safe waste disposal procedures [3,4,5,6,7,8]. It is clear that green chemistry requires the use of environmentally benign reagents and solvents, and it is very crucial to recover and reuse the catalyst. One way to overcome the problem of recyclability of the traditional acid catalysts is to chemically anchor their reactive centers onto an inorganic solid carrier with large surface area to create new organic–inorganic hybrid catalysts [9]. In this type of solids, the reactive centers are highly mobile, similar to homogeneous catalysts and at the same time, they have the advantage of recyclability like heterogeneous catalysts. In this view, several types of solid sulfonic acid functionalized silica (both amorphous and ordered) have been synthesized and applied as an alternative to traditional sulfonic acid resins and homogeneous acids in catalyzing chemical transformations [3,10,11]. Despite the attractiveness of these reagents, to the best of our knowledge, there is no report on the application of these catalysts in quinoxaline synthesis. Following the line of our studies on the design and application of solid catalysts in chemical transformations [12,13,14,15,16], we wish to describe herein, the preparation of silica bonded *S*-sulfonic acid (**SBSSA**) as illustrated in Scheme 1, which was then used as catalyst for the synthesis of quinoxaline derivatives. 

The preparation of quinoxaline and its derivatives plays an important role in organic synthesis [17,18]. These compounds constitute an important class of benzoheterocycles displaying a broad spectrum of biological activities, which have made them privileged structures in pharmacologically active compounds [19,20]. They have also been used as building blocks in the synthesis of organic semiconductors [21], rigid subunits in macrocyclic receptors or for molecular recognition [22], and as chemically controllable switches [23]. In general, these compounds can be produced via the condensation in organic solvents of 1,2-diamines with 1,2-dicarbonyl compounds under refluxing conditions with 34–85% yields for 2–12 h [24]. However, most of the traditional processes suffer from a variety of disadvantages such as pollution, high cost, poor chemical yields, requirements for long reaction times, and tedious work-up procedures, which limit their use as environmentally benign processes.

Some progress on the synthesis of quinoxaline derivatives has been reported in the literature, for example: the Bi-catalyzed oxidative coupling reaction [25], a tandem oxidation process using Pd(OAc)_2_ or RuCl_2_–(PPh_3_)_3_–TEMPO [26], and MnO_2_ [27], heteroannulation of nitroketene *N,S*-arylaminoacetals with POCl_3_ [28], cyclization of α-arylimino oximes compounds under refluxing condition in acetic anhydride [29]. Also, there are recent reports on the condensation of *o*-phenylene diamines and 1,2-dicarbonyl compounds in the presence of PbO [30], Zn[(L)-proline] in HOAc [31], copper chloride [32], montmorillonite K-10 [33], ([Hbim]BF4) [34], metal hydrogen sulfates [35], and oxalic acid [36]. 

**Scheme 2:**
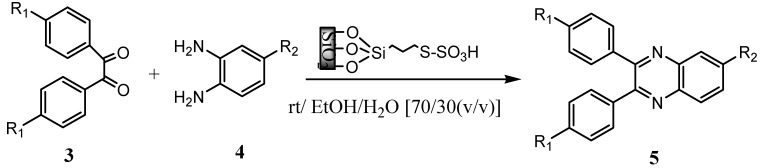
Synthesis of quinoxaline derivatives catalyzed by silica bonded *S*-sulfonic acid.

In connection with our studies on the preparation and applications of solid acids in organic transformations [12,13,14,15,16], we were interested in finding a simple and efficient method for the synthesis of quinoxaline derivatives under mild conditions using silica bonded *S*-sulfonic acid (**SBSSA**) as a catalyst (Scheme 2).

## 2. Results and Discussion

To begin this work the condensation reaction between benzil and *o*-phenylenediamine was employed as the model reaction to screen the best conditions (Table 1). As shown in the Table, condensation reaction in a mixture of ethanol/water [70/30 (v/v)] gave the best results in terms of time and yield, so we chose this solvent system for environmental acceptability.

**Table 1:** The influence of the solvent on the condensation reaction of *o*-phenylenediamine (1 mmol) and 1,2-dibenzylketone (1 mmol) catalyzed by **SBSSA** (3.4 mol%, 0.1 g).

Entry	Solvent	The amount of catalyst (g)	Time (min)	Yield (%)^a^
1	H_2_O	0.1	240	40
2	EtOH	0.1	12	97
3	CHCl_3_	0.1	60	63
4	CH_2_Cl_2_	0.1	60	59
5	EtOH/ H_2_O [30/ 70 (v/v)]	0.1	40	88
6	EtOH/ H_2_O [50/ 50 (v/v)]	0.1	30	90
7	EtOH/ H_2_O [70/ 30 (v/v)]	0.1	5	96
8	EtOH/ H_2_O [70/ 30 (v/v)]	0.03	25	75
9	EtOH/ H_2_O [70/ 30 (v/v)]	0.05	20	90
10	EtOH/ H_2_O [70/ 30 (v/v)]	0.15	5	96

^a^Isolated yield.

The effect of catalyst loading on the condensation reaction between benzil and *o*-phenylenediamine was also studied. This results show clearly that **SBSSA** is an effective catalyst for this condensation. Although this condensation could be accomplished with a lower catalyst loading 0.03 g of **SBSSA**, 0.1 g (3.4 mol%) of **SBSSA** per mmol of dicarbonyl compound was deemed optimum in terms of reaction time and isolated yield (Table 1, entries 7-10). The scope and generality of the present method was then further demonstrated by the condensation at room temperature of various 1,2-diaryldiketones with *o*-phenylenediamine derivatives using the optimized conditions {3.4 mol%, 0.1 g of **SBSSA** in EtOH/H_2_O [70/30 (v/v)]} and the results are presented in Table 2. The reaction proceeds very cleanly at this temperature and was free of side products. 

**Table 2:** Synthesis of quinoxaline derivatives catalyzed by **SBSSA** at room temperature.

Entry	Dicarbonyl compound	Diamine	Product	Time(min)	Yields(%)^a^
**5a**	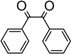	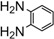	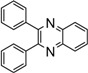	5	96
**5b**	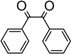	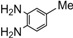	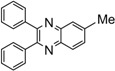	8	95
**5c**	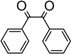	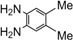	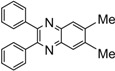	10	90
**5d**	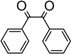	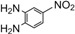	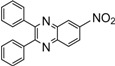	200	90
**5e**	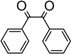	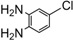	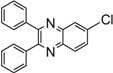	15	91
**5f**	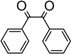	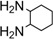	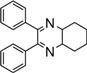	20	90
**5g**	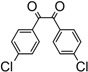	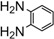	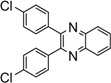	220	93
**5h**	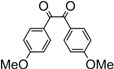	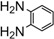	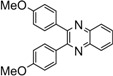	30	85
**5i**	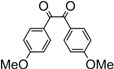	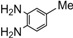	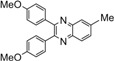	35	85
**5j**	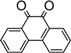	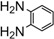	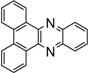	5	94
**5k**	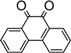	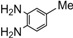	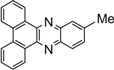	7	92
**5l**	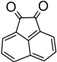	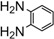	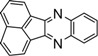	6	95
**5m**	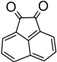	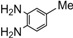	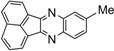	8	92
**5n**	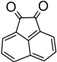	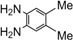	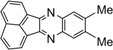	15	91
**5o**	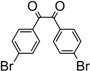	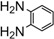	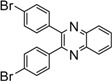	20	93
**5p**	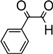	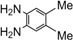	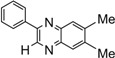	15	91

^a^Isolated yield.

It can easily be seen that the condensation reaction proceeded smoothly in the chosen solvent system and gave reasonable good to excellent yields, ranging from 85% to 96%. In the case of 1,2-diketones, either electron-withdrawing or electron-donating substituents (R_1 _= OMe, Cl, Br) on the aromatic ring gave slightly longer reaction times in comparison with R_1_= H (Table 2, entries 5g-i, 5o). However, for substituents on *o*-phenylenediamine (R_2_), electron-donating substituents (such as Me) reacted in shorter reaction times in comparison with electron-withdrawing groups (such as NO_2_). So, the order of the reactivity for condensation reaction was found to be: H > CH_3 _> Cl > NO_2_ (Table 2). 

**Figure 1:**
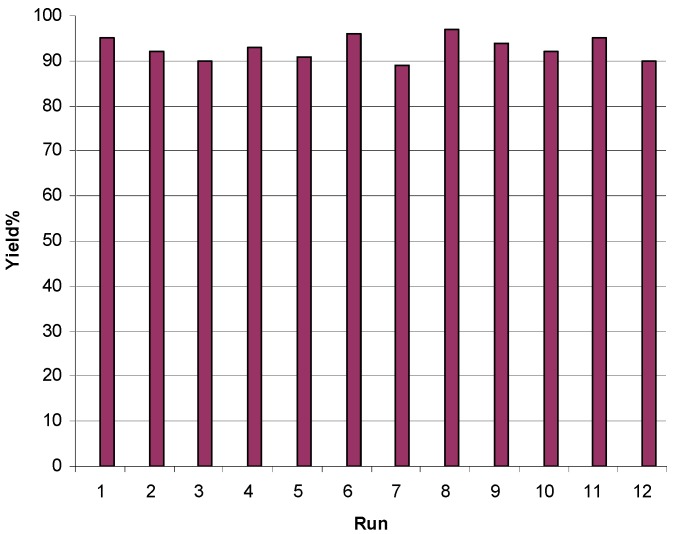
Recyclability of **SBSSA** (0.1 g) in the condensation reaction of *o*-phenylenediamine (1 mmol) with 1,2-dibenzylketone (1 mmol) at room temperature. Reaction time = 5 min.

The possibility of recycling the catalyst was examined. For this reason, the room temperature reaction of *o*-phenylenediamine and benzyl was studied in EtOH/H_2_O [70/30 (v/v)] in the presence of **SBSSA**. When the reaction was complete, the mixture was filtered, the residue was washed with warm ethanol and recycled catalyst was reused in the next reaction. No appreciable loss of catalytic activity was observed after twelve cycles (Figure 1).

**Scheme 3:**
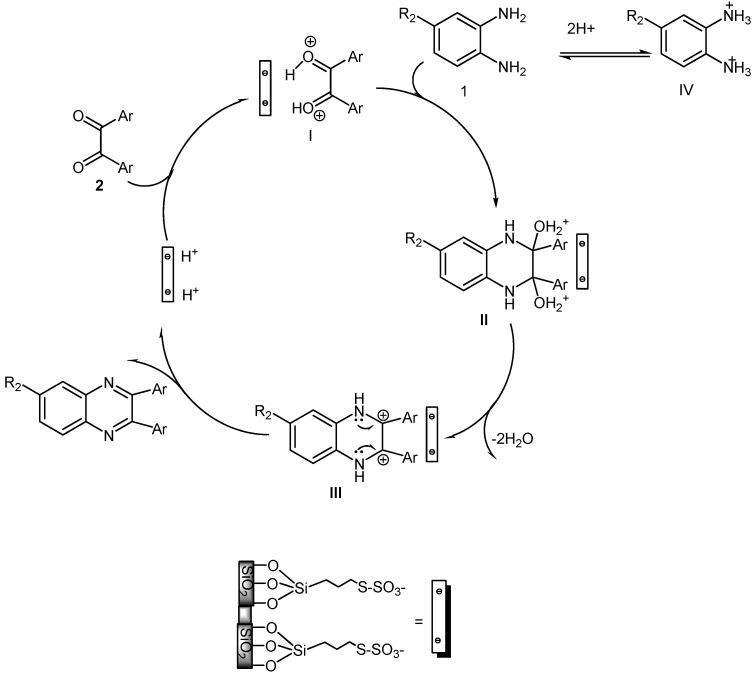
Proposed mechanism for the condensation reaction of 1,2-diamines with 1,2-dicarbonyl compounds catalyzed by **SBSSA.**

The proposed mechanism for the condensation reaction of 1,2-diamines with 1,2-dicarbonyl compounds in the presence of **SBSSA **is shown in Scheme 3.

The reaction is assumed to follow the regular mechanism of acid-catalyzed condensation reactions [26], with **SBSSA** acting as an acid in the protonation of the diketone and also playing a role in promoting the dehydration to give a carbocationic intermediate as shown in Scheme 3: (i) coordination of a 1,2-dicarbonyl onto acid sites from **SBSSA**, followed by (ii) the nucleophilic attack on the carbonyl C providing intermediate **I**, (iii) dehydration to give a carbocation intermediate and (iv) elimination of a proton to give the quinoxaline product.

## 3. Experimental Section

### 3.1. General

Chemicals were purchased from Fluka, Merck and Aldrich. IR spectra were run on a Shimadzu Infra Red Spectroscopy FT-IR-8000. The ^1^H and ^13^C NMR was run on Bruker Avance (DRX 500 MHz and 400 MHz) instruments in CDCl_3_. Results are reported in ppm. Melting points were recorded on a SMP1 Melting Point apparatus in open capillary tubes and are uncorrected. Reaction progress was followed by TLC using silica gel SILG/UV 254 plates. Measurements of surface area and pore size distribution were made using the Brunauer–Emmet–Teller (BET) method in a Quanta Sorb machine. The products were characterized by comparison of their spectral and physical data with those reported in literature [25,26,27,28,29,30,31,32,33,34,35,36]. 3-Mercapto-propylsilica (**1**, MPS) was prepared according to the previous report [3].

### 3.2. Catalyst Preparation

To a magnetically stirred mixture of 3-mercaptopropylsilica (**1**, 5 g) in CHCl_3_ (20 mL), chlorosulfonic acid (1.00 g, 9 mmol) was added dropwise at 0 ˚C during 2 h. After the addition was complete, the mixture was stirred for 2 h until all HCl was removed from reaction vessel. The mixture was then filtered, washed with methanol (30 mL) and dried at room temperature to give the silica bonded functionalized sulfonic acid **2** (**SBSSA**) as a cream powder (5.22 g). Sulfur content of the samples by conventional elemental analysis was 16.12%. Typically a loading at ca. 0.35 mmol/g is obtained. On the other hand, when the washed **SBSSA** was placed in an aqueous NaCl solution, the solution pH dropped virtually instantaneously to pH ≈ 1.85, as ion exchange occurred between protons and sodium ions (proton exchange capacity: 0.34 mmol/g of **SBSSA**. Also, a BET surface area of 435 m^2^g^-1^ and a total pore volume 0.63 cm^3^g^-1^ were measured for the catalyst.

### 3.3. General procedure for the synthesis of quinoxalines

To a stirred solution of 1,2-phenylenediamine (1 mmol) and dicarbonyl compound (1 mmol) in EtOH/H_2_O (70/30 v/v) (2 mL) was added **SBSSA** (3.4 mol%, 0.1 g) and stirred at room temperature. The reaction was followed by TLC. After completion of the reaction, the mixture was filtered, and the remaining was washed with warm ethanol in order to separate catalyst. Then water (20 mL) was added to the filtrate, and was allowed to stand at room temperature for 1 h. During this time, crystals of the pure product were formed which were collected by filtration and dried. For further purification if needed, the products recrystallized from hot ethanol.

*2,3-Diphenylquinoxaline* (**5a**): mp 126-127 ˚C (lit.[31] 128-129 ˚C); ^1^H-NMR (500 MHz) δ: 7.40 (m, 6H), 7.58 (m, 4H), 7.83 (m, 2H), 8.23 (d, 2H, J= 8.0 Hz); ^13^C-NMR (125 MHz) δ: 128.70, 129.23, 129.65, 130.28, 130.37, 139.54, 141.68, 153.91.

*6-Methyl-2,3-diphenylquinoxaline* (**5b**): mp 116-117 ˚C (lit.[31] 117-118 ˚C); ^1^H-NMR (500 MHz) δ: 2.64 (s, 3H), 7.35 (m, 6H), 7.54 (m, 4H), 7.63 (d, 1H, J= 8.5 Hz), 8.03 (s, 1H), 8.14 (d, 1H, J= 8.5 Hz); ^13^C-NMR (125 MHz) δ: 22.01, 127.69, 128.31, 128.52, 128.88, 128.98, 129.89, 129.94, 132.68, 138.55, 138.68, 139.48, 140.81, 141.02, 152.47, 153.07.

*6,7-Dimethyl-2,3-diphenylquinoxaline* (**5c**): mp 172-174 ˚C (lit.[37] 172 ˚C); ^1^H-NMR (500 MHz) δ: 2.52 (s, 6H), 7.31-7.33 (m, 6H), 7.50 (dd, 4H, J_1_= 7.6 Hz, J_2_= 1.6 Hz), 7.92 (s, 2H); ^13^C-NMR (125 MHz) δ: 20.84, 128.61, 128.66, 128.93, 130.27, 139.84, 140.66, 140.92, 152.93.

*6-Nitro-2,3-diphenylquinoxaline* (**5d**): mp 192-193 ˚C (lit.[31] 193-194 ˚C); ^1^H-NMR (400 MHz) δ: 7.42 (m, 6H), 7.58 (m, 4H), 8.32 (d, 1H, J= 8.0 Hz), 8.57 (m, 1H), 9.10 (s, 1H); ^13^C-NMR (100 MHz) δ: 124.41, 126.74, 129.54, 129.61, 130.80, 130.93, 130.99, 131.07, 131.90, 139.17, 139.23, 141.07, 144.70, 148.96, 156.80, 157.43.

*6-Chloro-2,3-diphenylquinoxaline* [17] (**5e**): mp 115-116 ˚C; ^1^H-NMR (500 MHz) δ: 7.36-7.44 (m, 6H), 7.56-7.57 (m, 4H), 7.74 (dd, 1H, J_1_= 8.9 Hz, J_2_= 2.3 Hz), 8.14 (d, 1H, J= 8.9 Hz), 8.22 (d, 1H, J= 2.3 Hz); ^13^C-NMR (125 MHz) δ: 128.52, 128.76, 129.46, 129.54, 130.25, 130.29, 130.87, 131.37, 136.08, 139.11, 139.18, 140.15, 141.92, 154.03, 154.71.

*2,3-Diphenyl-4a,5,6,7,8,8a-hexahydroquinoxaline* (**5f**): mp 167-169 ˚C (lit.[37] 167 ˚C); ^1^H-NMR (500 MHz) δ: 1.46-1.50 (m, 2H), 1.67-1.70 (m, 2H), 1.93-1.96 (m, 2H), 2.54-2.57 (m, 2H), 2.88-2.90 (m, 2H), 7.25-7.29 (m, 4H), 7.31-7.34 (m, 2H), 7.42-7.44 (m, 4H); ^13^C-NMR (125 MHz) δ: 25.87, 33.956, 59.95, 128.45, 128.55, 129.88, 138.23, 160.10.

*2,3-Bis(4-chlorophenyl)quinoxaline* (**5g**): mp 195-196 ˚C (lit.[31] 195-196 ˚C); ^1^H-NMR (500 MHz) δ: 7.39 (dt, 4H, J_1_= 8.5 Hz, J_2_= 2.0 Hz), 7.51 (dt, 4H, J_1_= 8.5 Hz, J_2_= 2.0 Hz), 7.83 (dd, 2H, J_1_= 6.3 Hz, J_2_= 3.4 Hz), 8.20 (dd, 2H, J_1_= 6.3 Hz, J_2_= 3.4 Hz); ^13^C NMR (125 MHz) δ: 129.15, 129.63, 130.79, 131.61, 135.78, 137.69, 141.67, 152.34. 

*2,3-Bis(4-methoxyphenyl)quinoxaline* (**5h**): mp 148-150 ˚C (lit.[36] 148-150 ˚C); ^1^H-NMR (400 MHz) δ: 3.84 (s, 6H), 6.85 (d, 4H, J= 8.6 Hz), 7.47 (d, 4H, J= 8.6 Hz), 7.68 (m, 2H), 8.19 (m, 2H).

*2,3-Bis(4-methoxyphenyl)-6-methylquinoxaline* (**5i**): mp 129-131 ˚C (lit.[36] 129-131 ˚C); ^1^H-NMR (400 MHz) δ: 1.58 (s, 3H), 3.88 (s, 6H), 6.65 (d, 4H, J= 8.6 Hz), 7.05 (d, 4H, J= 8.6 Hz), 7.61 (d, 1H, J= 8.5 Hz), 7.90 (s, 1H), 8.09 (d, 1H, J= 8.5 Hz).

*Dibenzo[a,c]phenazine* (**5j**): mp 223-225 ˚C (lit.[38] 224.8-225.7 ˚C); ^1^H-NMR (400 MHz) δ: 7.51-7.66 (m, 6H), 8.10-8.13 (m, 2H), 8.33 (d, 2H, J= 8.0 Hz), 9.18 (d, 2H, J= 8.1 Hz); ^13^C-NMR (100 MHz) δ: 124.01, 127.35, 129.01, 130.51, 130.81, 131.41, 133.23, 143.23, 143.51.

*11-Methyldibenzo[a,c]phenazine* [35] (**5k**): mp 208-210 ˚C;^ 1^H-NMR (400 MHz) δ: 2.46 (s, 3H), 7.46-7.58 (m, 5H), 7.85 (s, 1H), 7.97 (d, 1H, J= 8.0 Hz), 8.32 (d, 2H, J= 8.0 Hz), 9.13-9.16 (m, 2H); ^13^C-NMR (100 MHz) δ: 23.20, 123.95, 127.15, 127.29, 128.92, 129.10, 130.01, 131.07, 131.20, 131.45, 131.49, 132.87, 133.06, 133.45, 141.41, 141.81, 142.72, 143.27, 143.29. 

*Acenaphtho[1,2-b]quinoxaline* (**5l**): mp 238-240 ˚C (lit.[38] 239.5-241.3 °C); ^1^H-NMR (400 MHz) δ: 7.55-7.65 (m, 4H), 7.89 (d, 2H, J= 8.4 Hz), 8.00-8.20 (m, 2H), 8.21 (d, 2H, J= 6.8 Hz); ^13^C-NMR (100 MHz) δ: 122.96, 129.78, 130.36, 130.59, 130.74, 131.10, 132.92, 137.60, 142.39, 155.19.

*9-Methylacenaphtho[1,2-b]quinoxaline* [35] (**5m**): mp >300 ˚C; ^1^H-NMR (500 MHz) δ: 2.60 (s, 3H), 7.55 (d, 1H, J= 8.25 Hz), 7.79 (t, 2H, J= 7.5 Hz), 7.95 (s, 1H), 8.03-8.07 (m, 3H), 8.35 (t, 2H, J = 6.3 Hz); ^13^C-NMR (125 MHz) δ: 22.19, 121.97, 122.12, 129.01, 129.03, 129.21, 129.53, 129.60, 129.75, 130.39, 131.72, 132.44, 136.68, 140.06, 140.10, 141.73, 153.76, 154.48. 

*9,10-Dimethylacenaphtho[1,2-b]quinoxaline* (**5n**): mp 304-306 ˚C (lit.[36] 304-306 ˚C); ^1^H-NMR (400 MHz) δ: 2.51 (s, 6H), 7.78 (m, 2H), 7.89 (s, 2H), 8.03 (m, 2H), 8.34 (m, 2H); ^13^C-NMR (100 MHz) δ: 20.3, 121.5, 127.8, 128.0, 128.6, 128.9, 129.1, 139.5, 140.0, 148.5, 153.3.

*2,3-Bis(4-bromophenyl)quinoxaline* (**5o**): mp 192-194 ˚C (lit.[34] 194-195 ˚C); ^1^H-NMR (500 MHz) δ: 7.41 (dt, 4H, J_1_= 8.5 Hz, J_2_= 2.0 Hz), 7.51 (dt, 4H, J_1_= 8.5 Hz, J_2_= 2.0 Hz), 7.79 (dd, 2H, J_1_= 6.5 Hz, J_2_= 3.5 Hz), 8.15 (dd, 2H, J_1_= 6.5 Hz, J_2_= 3.5 Hz); ^13^C-NMR (125 MHz) δ: 124.13, 129.64, 130.84, 131.86, 132.12, 138.12, 141.68, 152.34.

*6,7-Dimethyl-2-phenylquinoxaline* (**5p**): mp 127-129 ˚C (lit.[32] 128-129 ˚C); ^1^H-NMR (500 MHz) δ: 2.55 (s, 6H), 7.55 (t, 1H, J= 7.1 Hz), 7.59 (t, 2H, J= 7.1 Hz), 7.89 (s, 1H), 7.95 (s, 1H), 8.21 (d, 2H, J = 7.2 Hz), 9.26 (s, 1H); ^13^C-NMR (125 MHz) δ: 20.75, 20.78, 127.80, 128.59, 129.10, 129.48, 130.24, 137.58, 140.50, 141.02, 141.18, 141.66, 142.83, 151.42. 

## 4. Conclusions

In summary, this work shows that silica bonded *S*-sulfonic acid, prepared from commercially available and relatively cheap starting materials by a simple transformation, efficiently catalyzes the room temperature condensation reactions between 1,2-diamino compounds and 1,2-dicarbonyl compounds to give quinoxaline derivatives in good to excellent yield. It could also be recovered and reused for more than twelve reaction cycles without noticeable loss of reactivity.
